# Comparative omics and feeding manipulations in chicken indicate a shift of the endocrine role of visceral fat towards reproduction

**DOI:** 10.1186/s12864-018-4675-0

**Published:** 2018-04-26

**Authors:** Susanne Bornelöv, Eyal Seroussi, Sara Yosefi, Sharon Benjamini, Shoval Miyara, Mark Ruzal, Manfred Grabherr, Nima Rafati, Anna-Maja Molin, Ken Pendavis, Shane C. Burgess, Leif Andersson, Miriam Friedman-Einat

**Affiliations:** 1 Science for Life Laboratory, Department of Medical Biochemistry and Microbiology, Uppsala University, SE-751 23 Uppsala, Sweden; 2 Present Address: Wellcome Trust Medical Research Council Cambridge Stem Cell Institute, University of Cambridge, Cambridge, CB2 1QR UK; 3 Agricultural Research Organization, Volcani Center, Rishon LeZion, Israel; 4 Robert H. Smith Faculty of Agriculture, Food and Environment, Hebrew University of Jerusalem, 76100 Rehovot, Israel; 5 Bioinformatics Infrastructure for Life Sciences, Uppsala University, Uppsala, Sweden; 6 College of Agriculture and Life Sciences, University of Arizona, Tucson, USA; 7 Department of Animal Breeding and Genetics, Swedish University of Agricultural Sciences, SE-750 07 Uppsala, Sweden; 8 Department of Veterinary Integrative Biosciences, College of Veterinary Medicine and Biomedical Sciences, Texas A&M University, College Station, TX 77843–4458 USA

**Keywords:** Chickens, Adipose tissue, Adipokines, PTEN-pathway, *Adipolin*, *SFTPA1*, *TNF*, *PLIN1*, Yolk proteins, RNA-seq, Mass spectrometry

## Abstract

**Background:**

The mammalian adipose tissue plays a central role in energy-balance control, whereas the avian visceral fat hardly expresses leptin, the key adipokine in mammals. Therefore, to assess the endocrine role of adipose tissue in birds, we compared the transcriptome and proteome between two metabolically different types of chickens, broilers and layers, bred towards efficient meat and egg production, respectively.

**Results:**

Broilers and layer hens, grown up to sexual maturation under free-feeding conditions, differed 4.0-fold in weight and 1.6-fold in ovarian-follicle counts, yet the relative accumulation of visceral fat was comparable. RNA-seq and mass-spectrometry (MS) analyses of visceral fat revealed differentially expressed genes between broilers and layers, 1106 at the mRNA level (FDR ≤ 0.05), and 203 at the protein level (*P* ≤ 0.05). In broilers, Ingenuity Pathway Analysis revealed activation of the PTEN-pathway, and in layers increased response to external signals. The expression pattern of genes encoding fat-secreted proteins in broilers and layers was characterized in the RNA-seq and MS data, as well as by qPCR on visceral fat under free feeding and 24 h-feed deprivation. This characterization was expanded using available RNA-seq data of tissues from red junglefowl, and of visceral fat from broilers of different types. These comparisons revealed expression of new adipokines and secreted proteins (*LCAT*, *LECT2*, *SERPINE2*, *SFTP1*, *ZP1*, *ZP3*, *APOV1*, *VTG1* and *VTG2*) at the mRNA and/or protein levels, with dynamic gene expression patterns in the selected chicken lines (except for *ZP1*; FDR/*P* ≤ 0.05) and feed deprivation (*NAMPT*, *SFTPA1* and *ZP3*) (*P* ≤ 0.05). In contrast, some of the most prominent adipokines in mammals, leptin, *TNF*, *IFNG*, and *IL6* were expressed at a low level (FPKM/RPKM< 1) and did not show differential mRNA expression neither between broiler and layer lines nor between fed vs. feed-deprived chickens.

**Conclusions:**

Our study revealed that RNA and protein expression in visceral fat changes with selective breeding, suggesting endocrine roles of visceral fat in the selected phenotypes. In comparison to gene expression in visceral fat of mammals, our findings points to a more direct cross talk of the chicken visceral fat with the reproductive system and lower involvement in the regulation of appetite, inflammation and insulin resistance.

**Electronic supplementary material:**

The online version of this article (10.1186/s12864-018-4675-0) contains supplementary material, which is available to authorized users.

## Background

The chicken was domesticated from its wild ancestor, the red junglefowl, about 6000 years ago. Since then, a variety of chicken breeds were created by geographical isolation and selection for desired traits [1]. Diversifying selection of chickens for high growth rate and high egg production subsequently led to distinct phenotypes, characterized as broiler and layers [2]. Thus, broiler and layer chickens provide a model system to study the genetic basis of these traits.

As a result of the breeding strategy, modern broilers reach about five times higher body weight (BW) during the fast-growing period before sexual maturation, compared to layers, and about four times higher body weight compared to the broiler strains of only six decades ago, which marked the beginning of intensive commercial breeding. Layer hens, on the other hand, produce almost one egg per day during the first year of lay, and in total more than 2.5 fold more eggs than broiler hens under feed restriction [3]. Fertility and hatchability of the fertilized eggs are also higher in layers compared to broilers [4]. Much of the difference in reproduction efficiency between the broiler and layer hens is associated with their ovarian morphology. Under ad libitum feeding, the ovary of a broiler breeding hen contains about twice as many yellow follicles than are present in a layer hen, resulting in disrupted hierarchy and hampered reproduction efficiency [5]. In addition, broilers suffer from higher predisposition to pulmonary arterial hypertension, heart failure (ascites), impaired immunity, and higher insulin resistance compared to layers [6, 7]. Strong feed restriction regiments, used in the commercial growing of broiler breeders, greatly improve these metabolic disorders and correct the ovarian morphology [5]. In layer hens, ad libitum feeding does not harm their production traits. However towards old age, layers can develop diseases such as fatty liver hemorrhagic syndrome, osteoporosis and hypocalcemia [8]. We hypothesized that the adipose tissue may have a role in these traits, and that gene expression profiling on both the mRNA and protein levels may shed light on the regulatory mechanisms underlying these phenotypes in birds.

Adipose tissue stores excess triglycerides and releases them during periods of negative energy balance. In addition, since the discovery of the mammalian leptin in 1994 [9], it has become evident that the adipose tissue has a central endocrine role in mammals [10], operating by secretion of leptin and other signaling molecules, named adipokines [11]. In mammals, adipokines are implicated in the regulation of many physiological processes, such as appetite and satiety, fat distribution, inflammation, blood pressure, hemostasis, insulin sensitivity, reproduction, immune response, and endothelial function [12, 13]. However, in birds much less is known about the expression of these adipokines and their endocrine role [14].

Leptin, the key adipokine in the endocrine activity of the fat tissue in mammals, has only recently been identified and characterized in birds [15–18] and its expression was detected in a variety of tissues, but with apparent insignificant expression in the adipose tissue [15, 16, 18]. We also recently identified an additional key adipokine, *TNF* (also called *TNF-α*), previously missing in chicken [19]. Unlike leptin, *TNF* has a similar expression profile in chickens compared to mammals, including expression in the adipose tissue. These new findings emphasize the need for a combined analysis of global gene expression study and the analysis of candidate genes for unraveling the endocrine role of visceral adipose tissue in birds.

## Results

### Phenotypic characterization

**Fig. 1:**
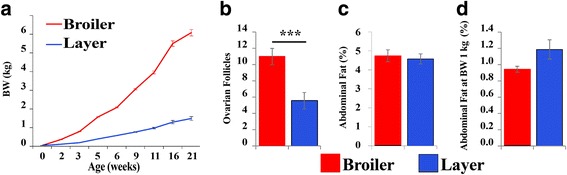
Broiler and layer females differ in growth rate and reproduction efficiency, but not in the accumulation of the visceral fat. **a** BW measurements were obtained in a follow-up experiment of broiler breeders and layer hens, grown together from hatch with free access to food (*n* = 50 for each bird’s type; *P* < 0.001 at all ages including the day of hatch). **b** The number of large yellow follicle of ≥8 mm were counted in the ovary of the broiler and layer females at the first week of lay (4 month of age; *n* = 10 for each group). **c** Percentages of visceral fat (fat weight/live BW) were analyzed in the same birds shown in (**b**). **d** Percentages of visceral weight of broiler and layer chickens were measures when reaching 1 Kg BW. n = 10 for each bird’s type. *** P < 0.001

In the present study, female broiler breeder (Cobb) and layer (Leghorn, Lehmann) chickens were grown together under the same conditions with free access to food and water. At the day of hatch, the body weight (BW) of broiler and layer chicks differed by only about 10% (42.4 ± 0.4 and 38.3 ± 0.7 g, respectively). The difference in BW between the two strains, observed in the following weeks and at sexual maturation (21 weeks of age, broilers 6038 ± 113 g and layers 1503 ± 100 g; Fig. 1a), was huge as expected [6]. Analysis of ovarian morphology revealed an excessive number of ovarian follicles (about 1.6-fold) in broilers compared to layers (Fig. 1b), which was also expected [5]. Abdominal fat accumulation, measured at sexual maturation, showed no difference when calculated as the percent of BW (Fig. 1c). This observation, which is in contrast to previous publications [20], likely reflects the recent strong selection against excessive fat deposition in commercial broilers. Since this is the first demonstration that under a free feeding regiment, broiler and layer hens have similar relative body fat accumulation, we also compared abdominal fat accumulation at a weight of 1 kg (about 3 and 11 weeks of age for broilers and layers, respectively; Fig. 1a). Also at the same BW, no significant difference in visceral fat accumulation was observed (Fig. 1d).

In short, the broiler and layer hens used in the current study differed in the rate of body growth and ovarian morphology, reflecting the well-known higher body growth rate in broilers and higher egg production in layers [6]. However, the modern broiler hens cannot be regarded as a model of obesity as suggested before [21].

### Differential gene expression analysis in visceral fat by RNA-seq

**Fig. 2:**
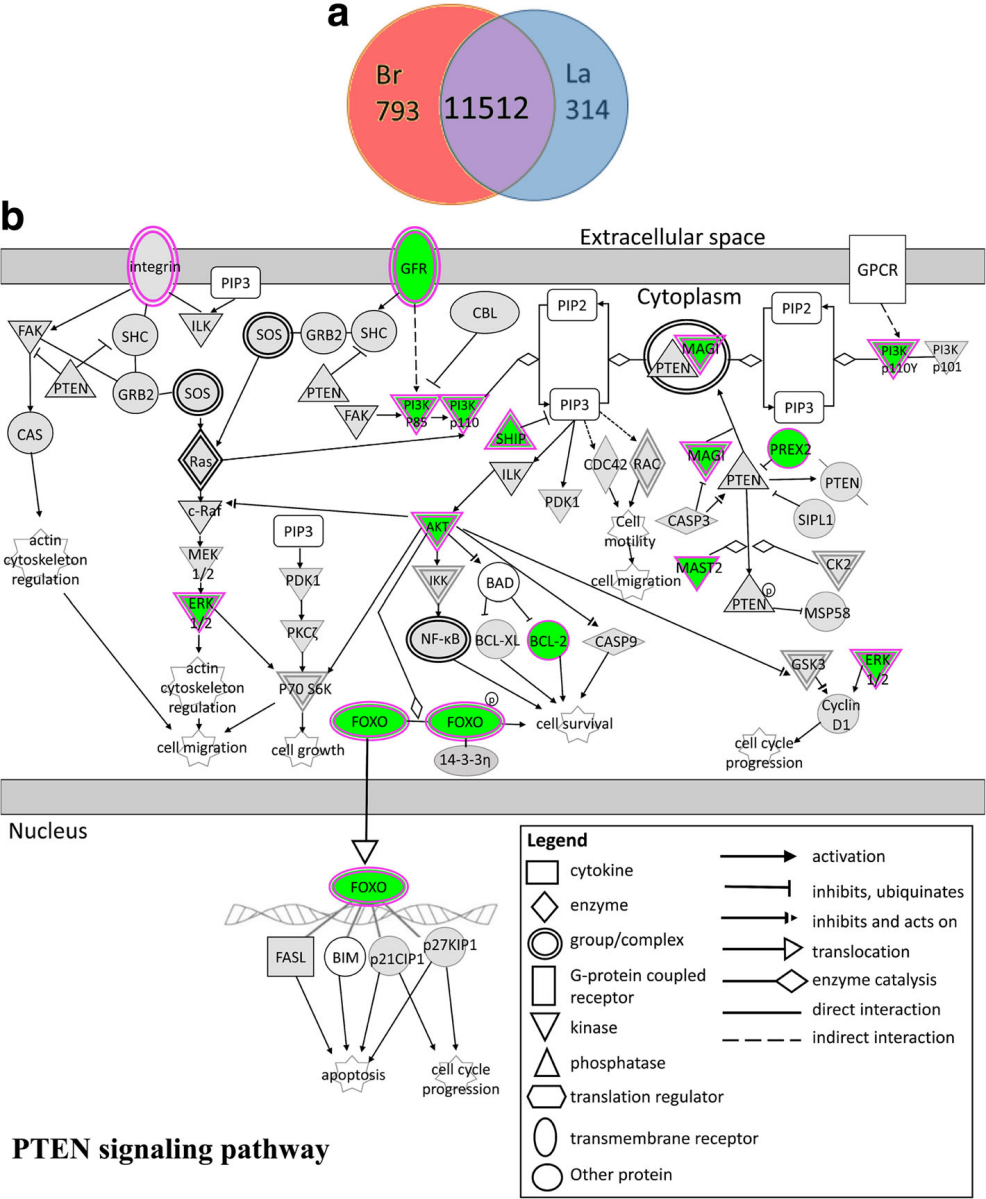
Differential gene expression in visceral abdominal fat of broiler and layer females. **a** Venn Diagrams depicts the number of transcripts differentially expressed in broilers (Br) and layers (La) or not differentially expressed (FDR ≤ 0.05; absolute fold change ≥1.5; *n* = 3 birds per strain). **b** The *PTEN* pathway was selected by Ingenuity software as the dominant pathway enriched in broilers compared to layers (Z-score = 3.6; ratio = 0.15; *P* < 0.001; Additional file 1: Table S3)

To identify differentially expressed genes in visceral fat of broiler and layer chickens, we performed RNA-seq analysis on samples from three female broiler and three female layer chickens at the first week of lay (Additional file 1: Table S1). Sequence data were mapped to the reference genome assembly Galgal4 (GCA_000002315.2) and annotations were adapted to Galgal5 (GCA_000002315.3). Comparative analysis with *P*-values adjusted for false discovery rate (FDR) using edgeR revealed 1107 differentially expressed genes at FDR ≤ 0.05 and absolute fold change ≥1.5 (Fig. 2a; Additional file 1: Table S2). The set of differentially expressed gene was submitted to Ingenuity Pathway Analysis, using gene annotations of the corresponding human genes. A prominent elevation of the PTEN pathway was observed in broilers [Z-score 3.6; ratio 0.15; *P* < 0.001; Additional file 1: Table S3]. This pathway encompasses signals from growth factor receptors on the cell surface to transcription factors through inhibition of the PI3K/Akt signaling, which regulate cell proliferation, migration and survival [22], as well as glucose uptake and insulin sensitivity [23, 24]. As schematically demonstrated in Fig. 2b, the *phosphatase and tensin homolog deleted on chromosome 10* (*PTEN*) mRNA was not differentially expressed between broilers and layers. However, transcripts implicated in the PTEN antagonistic signaling pathway PI3K/AKT and its downstream activities were lower in broilers (FDR ≤ 0.05; absolute fold change ≥1.5; *MAST2, PIK3CA, MAPK1, FLT1, PIK3R1, FLT4, BMPR2, PREX2, CNKSR3, BCL2, SYNJ1, FOXO1, BMPR1A, PIK3CG, IGF1R, AKT3, KDR, ITGA4*). Loss of *PTEN* leads to constitutive insulin sensitivity and obesity, in addition to high susceptibility to cancer [25]. Therefore, the enrichment of increased expression of members of the PTEN pathway in broilers may explain their higher insulin resistance [7].

In layers, pathways that involve PI3K/AKT signaling were enriched as a mirror image of the enrichment of PTEN pathway in broilers. These include response to signaling by nitric oxide, IL8, relaxin, renin-arginine, leukocyte extravasation, HGF, VEGF, macropinocytosis, GH, NFAT, TEC kinase, eNOS, and NFkB (*P* < 0.001; Z-score ≤ − 2; Additional file 1: Table S3). Taken together, the pathway analysis suggests a biological difference of the metabolic activity in visceral fat in broilers and layers, stemming from a variation in the balance between two prominent signaling pathways: PTEN and PI3K/AKT. This difference correlates with the higher insulin resistance in broilers [7].

### mRNA expression profile of adipokine and fat secreted proteins

**Fig. 3:**
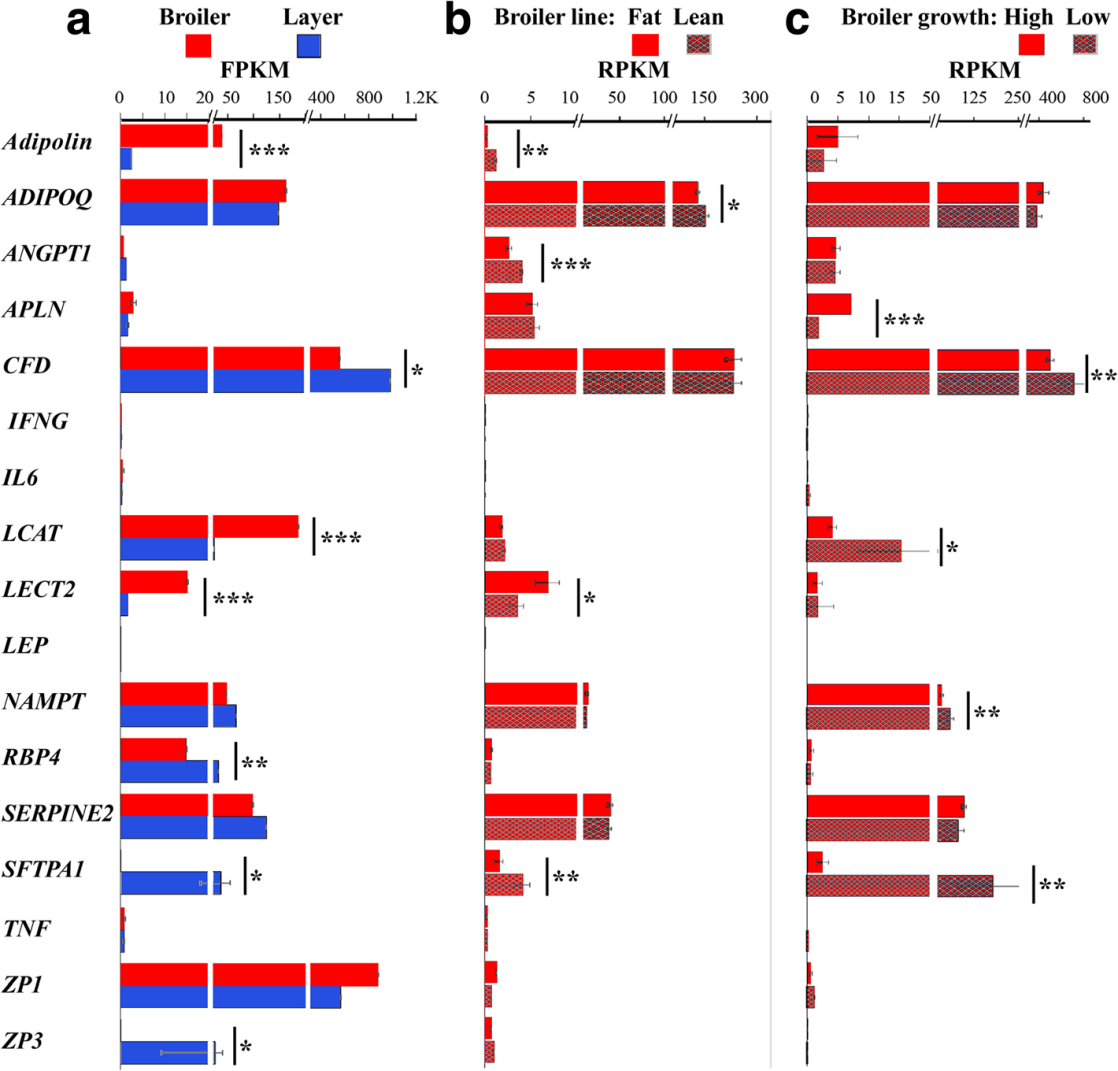
Expression profile of selected adipokines in visceral fat. **a** Expression profile in broiler and layer females was depicted from our RNA-seq data (Additional file 1: Table S2) calculated as fragments per kilobase of transcript per million mapped reads (FPKM) using edgeR and FDR value with threshold 0.05. Values are expressed as means ± SD. n = 3 in each group. *, FDR ≤ 0.05; **, FDR ≤ 0.01. *APLN, IFNG*, *Leptin* (*LEP*), *SFTPA1*, *TNF*, and *ZP3*, which are missing from the RNA-seq table either due to missing annotation in Galgal5 or low expression level, were searched manually in the RNA-seq data. *P* values of these transcripts were calculated by student t-test or rank transformed, in cases of lack of normal distribution, and expressed as means ± SE. **b** and **c** Expression profiles obtained by blast search using GenBank available experiments in two juvenile broiler experiments: fat and lean lines or high and low growth lines, respectively. The data was calculated as reads per kilobase of transcript per million mapped reads (RPKM). *P* values were calculated by student t-test and expressed as means ± SE. accession no. are detailed in the Material and Methods section

Our main interest in adipose tissue relies on its known central role in the control of energy balance in mammals, mediated through secretion of adipokines with afferent metabolic signaling [11]. To assess if there is a similar role of the adipose tissue in chickens, we focused on fat secreted proteins in the broiler and layer hens (Fig. 3a). The most prominent adipokines in mammals implicated in regulation of appetite, insulin sensitivity and inflammation including leptin, tumor necrosis α (*TNF*) interferon-γ (*IFNG*), and interleukin-6 (*IL6*) were expressed at low levels (below 1 fragments per kilobase of transcript per million mapped reads; FPKM) in both broilers and layers. Among the other characterized transcripts of known adipokines, the highest differential expression between broilers and layers was observed for the anti-inflammatory adipokine in mammals, adipolin (*FAM132A*, also known as *C1QDC2* and *CTRP12*; [26]), which had about 14.7-fold higher level of mRNA in broilers (FDR = 6.5 × 10^− 26^). Two known hepatokines, lecithin cholesterol acyltransferase (*LCAT;* [27]) and cell-derived chemotaxin 2 (*LECT2;* [28])*,* were also expressed at a higher level in broilers (7.8-fold, FDR = 5 × 10^− 8^ and 8.9-fold, FDR = 4 × 10^− 8^, respectively). In layers, two known adipokines, retinol-binding protein 4 (*RBP4*) and adipsin (*CFD*), and two newly identified transcripts in the adipose tissue, zona pellucida protein 3 (*ZP3*) and pulmonary surfactant-associated protein A1 (*SFTPA1*) were expressed at a higher level (FDR/*P* ≤ 0.05; with fold differences of about 1.8, 2.1, 170 and 500, respectively).

Other transcripts coding for secreted proteins that we identified in visceral fat of broilers and layers were not differentially expressed. These included two known adipokines, adiponectin (*ADIPO-Q)* and apelin (*APLN*), nicotinamide phosphoribosyl transferase (*NAMP1*, also known as visfatin), and two transcripts characterized in the adipose tissue for the first time, glia-derived nexin (*SERPINE2*) and zona pellucida protein 1 (*ZP1*).

To assess the possible physiological implications of the expression profile described in Fig. 3a, we explored the expression of these genes also in RNA-seq data from visceral fat of two other chicken models that are available in GenBank. These include two broiler lines, divergently selected for fat and lean phenotype (FL-Br and LL-Br, respectively; [29]) and two other broiler lines divergently selected for high and low body growth (HG-Br and LG-Br; [30]). Both of these datasets represented mRNA expression in visceral fat of 7 weeks old males. At this age, FL-Br had on average a 2.6-fold higher accumulation of visceral fat than LL-Br and the same BW [29], while HG-Br had on average a 19.6-fold higher visceral fat accumulation than LG-Br and a 3.2-fold higher BW [30]. Thus, also when normalized to BW, both FL-Br and HG-Br showed higher visceral fat accumulation compared to their respective controls (2.6- and 6.1-fold, respectively), with the HG-Br also differing in higher BW. Blast analyses of these datasets using the sequences of the selected fat secreted proteins as baits are shown in Figs. 3b & 3c. Similarly to the broiler-layer comparison, transcripts of the prominent adipokines in mammals, leptin, *TNF*, *IFNG*, and *IL6* were low and did not show statistically significant differential expression. This suggests that leptin, *TNF*, *IFNG*, and *IL6* are not implicated in the regulation of the pronounced phenotypic differences between these chicken lines. A general similarity in FPKM/reads-per-kilobase-of-transcript-per-million-mapped-reads (RPKM) levels of expression was observed between the sexually mature broilers and the juvenile broiler lines also for the prominently expressed genes *ADIPOQ*, *APLN*, *NAMPT* and *SERPINE2,* with a maximum of a 3-fold difference. Therefore, it is striking that the *ZP1* mRNA was expressed at about an 800-fold lower level in the juvenile broilers compared to the sexually mature broiler hens. This observation is compatible with the expected role of *ZP1* in female reproduction, and with the report by Hanafy et al., showing that expression of *ZP1* in the liver of quail is induced by sexual maturation and by treatment with estrogen [31]. It is likely that the ~ 10-fold lower mRNA levels of *RBP4*, *LCAT* and adipolin in the juvenile broiler lines compared to their expression in mature broiler females are also related to sexual maturation and reproduction. For *LCAT*, this has been indicated by Noble et al., which showed that plasma LCAT levels are induced by sexual maturation of layer hens and implicated in transporting HDL triglycerides to the egg yolk [32].

The search in the FL-Br and LL-Br data indicated that similarly to mammals, expression of adipolin and *ADIPOQ* correlates with lean phenotype. However, adipolin and *ADIPOQ* mRNA were expressed in HG and LG-Br at a similar mRNA level and was ~ 15-fold higher in broilers compared to layers, suggesting an additional role in promoting body growth. The proangiogenic factor *ANGPT1* [33] was expressed at a higher mRNA level in LL-Br compared to HF-Br, which is compatible with the known impairment of angiogenesis in obesity [34]. Its non-differential expression in visceral fat of the HG-Br compared to LG-Br suggests that selection for normal expression of *ANGPT1* was important to achieve high growth rate due to its critical role in angiogenesis [33]. Comparing to LG-Br, a high level of transcription was detected in HG-Br for the atheroprotective peptide *APLN* (*P* = 6e^− 9^), which enhances glucose utilization and improves insulin sensitivity. *CDF* mRNA, which was expressed at a lower level in broilers compared to layers, was also expressed at lower levels in HG-Br compared to LG-Br, associating this gene with the restricted growth rate of chickens. *LCAT, NAMP1,* and *SFTPA1* mRNAs were also found at a lower level in HG-Br (*P* ≤ 0.05). Among them, the *SFTPA1* mRNA expression was strikingly 72-fold higher in LL-Br compared to FL-Br (*P* = 0.006), suggesting a role in restricting the rate of body growth and fat deposition. Altogether, the identification of new adipokines and secreted proteins by RNA-seq analysis of visceral fat of broilers and layers (*LECT2, LCAT, SERPINE2, SFTPA1, ZP1*, and *ZP3*), was confirmed also by the search in available RNA-seq data of juvenile broiler lines. Except for *SERPINE2* and *ZP1*, the other mRNAs were differentially expressed between broiler and layer chickens and/or the experimentally selected lines of broiler. In contrast, some of the critical adipokines in mammals (leptin, *TNF*, *IFNG* and *IL6*) were lowly expressed and did not show differential expression between lines *(P* > 0.05).

### Expression profiling of the newly identified fat secreted proteins *LECT2, LCAT, SERPINE2, SFTPA1, ZP1*, and *ZP3* in tissues of red junglefowl

**Fig. 4:**
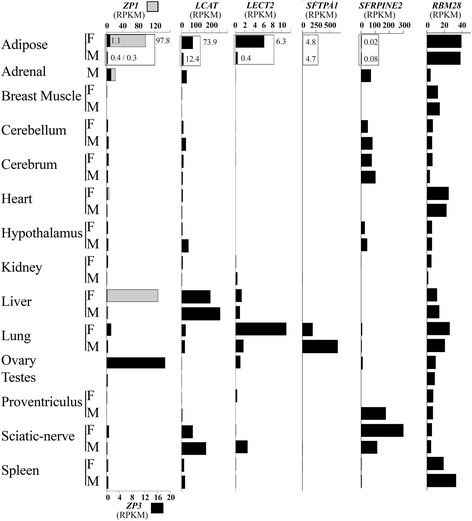
Expression profile of genes characterized in visceral fat for the first time and a control gene (*RBM28*), using the available RNA-seq dataset of red junglefowl. Full-length cDNAs of the indicated genes were used as baits for blast search. Accession no. are detailed in the Material and Methods section. Gray bars indicate expression of *ZP1,* black bars indicate expression of *ZP3*, *LCAT*, *LECT2*, *SFTPA1*, *SERPINE2*, and *RBM28*

To more accurately estimate the tissue specificity of the secreted proteins that have not been previously observed in visceral fat of chicken, we used the available RNA-seq datasets of a variety of tissues from 2 years old red junglefowl [accession number ERA252218], which included 137 RNA-seq experiments from a male and a female bird (Fig. 4).

The expression of *ZP1* and *ZP3,* which in mammals are synthetized solely by growing oocytes [35, 36], showed that *ZP1* was dominantly expressed in both visceral fat and liver, while *ZP3* mRNA was dominant in the ovary. The specificity of this expression to the female red junglefowl demonstrates the relevance of this expression to female reproduction. In other oviparous vertebrates, these proteins are found in the liver either in addition or instead of the ovary [37, 38]. In birds, extra gonadal expression of *ZP1* was reported in the liver of quail [31], where it was also shown that intravenously injected *ZP1* incorporates into the perivitelline membrane [39]. Therefore, our finding of *ZP1* and *ZP3* expression in visceral fat suggests a chicken/bird specific mechanism of metabolic regulation of fertility.

The expression of the known hepatokines *LCAT* and *LECT2* [48, 49] in the adipose tissue was also specific to the female red junglefowl, supporting their relevance to female reproduction. Implication of *LCAT* in female reproduction was suggested by Hengstschlager-Ottnad et al. [40], showing dynamic changes of *LCAT* expression in the liver and brain upon sexual maturation of laying chickens. Interestingly, although expected, *LCAT* was also transcribed in the liver [41], *LECT2* mRNA was unexpectedly observed in the female lung. To verify that the sequence we used in this search is the genuine chicken *LECT2*, we compared local synteny of the human and chicken *LECT2*, and found the same neighboring genes *(IL9* and *TGFB1*). Therefore, it seems that the chicken *LECT2* gene has different function compared to its mammalian ortholog, including a role in the adipose tissue. As a control for the gender-specific expression, we searched the same datasets with the chicken *RBM28* bait [42]. *RBM28* and other housekeeping genes that we have used in a similar search indicated that the gender specific pattern of *ZP1*, *ZP3*, *LCAT*, and *LECT2* mRNA expression, is specific to these genes.

The pulmonary *SFTPA1* and glial *SERPINE2* genes in red junglefowl (Fig. 4) showed a relatively low level of expression of *SFTPA1* in visceral fat and no significant expression for *SERPINE2* (about 5 and below 0.1 RPKM, respectively). *SFTPA1* was predominantly expressed in the lung and *SERPINE2* in brain tissues, sciatic nerve, adrenal and proventriculus. These expression patterns fit with the known essential role of *SFTPA1* in the structure and function of the pulmonary alveoli [43], and the pleotropic role of *SERPINE2* in nerve and other cell types [44]. Nevertheless, our finding of relatively high expression levels of *SFTPA1* and *SERPINE2* in visceral fat of commercial chickens (Fig. 3) suggests their recruitment to expression in visceral fat of chickens possibly due to selective breeding in the commercial lines. Although *SFTPA1* and *SERPINE2* were not implicated in adipose tissue before, their characterized roles as a phospholipid binding protein (SFTPA1; [43]), extra cellular matrix modification and inhibition of blood coagulation (SERPINE2; [44]) are compatible with a metabolic role in the adipose tissue. Altogether, the expression profile analysis in red junglefowl revealed recruitment of gene expression to the adipose tissue in chickens for *ZP1*, *ZP3*, *LCAT* and *LECT2*, either in addition (*ZP3* and *LCAT*) or instead (*ZP1* and *LECT2*) of their known expression pattern in mammals. The higher expression of *Zp1*,*Zp3*, *LCAT* and *LECT2* in visceral fat of the female compared to the male, further supports their implication in female reproduction. For *SFTPA1* and *SEPRINE2* the study suggests a putative role in the phenotypic difference between wild and domestic chicken lines.

### Effect of 24 h feed deprivation on adipokine expression

**Fig. 5:**
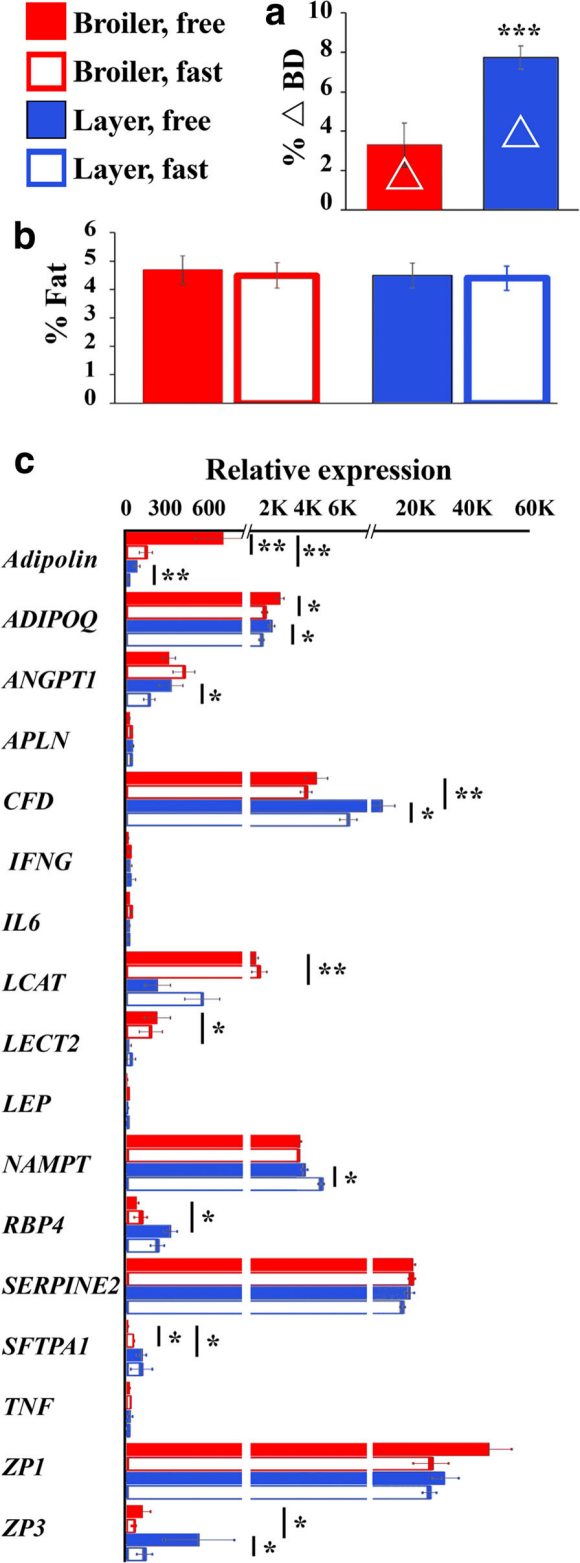
Response of adipokine expression to feed deprivation. **a** The effect of 24 h feed deprivation (Fast) on BW of the broiler and layer chickens was calculated as the percent of BW change of the initial BW (before feed deprivation). **b** The effect of the treatment on relative weight of the visceral fat. **c**. qPCR analysis of expression of the indicated adipokines. Vertical lines represent ± SE, *n* = 7 for each bird’s type and treatment, in each of the two biological repeats. Significance was calculated using students t test and denoted as *, *P* ≤ 0.05; **; *P* ≤ 0.01; ***, *P* ≤ 0.005. Long and short horizontal lines indicate comparison between broilers and layers both at free feeding, or between free and deprived feeding, respectively. Primers are listed in Additional file 2: Table S4

To further estimate the possible implication of the secreted proteins listed in Fig. 3 in the control of energy balance, we explored the effect of feed deprivation on their transcription. Broiler and layer females were grown as described in Fig. 1, but, immediately before tissue sampling, half of the birds of each type were subjected to a 24 h feed deprivation. Relative BW loss following this feed deprivation was much higher in layers compared to broilers when calculated as percent BW loss (Fig. 5a), reflecting their higher energy expenditure and lower feed efficiency [45]. Under this fasting condition, there was no significant (*P* ≤ 0.05) effect on the relative weight of the visceral fat between the two groups (Fig. 5b), suggesting that the liver glycogen stores were the dominant source of energy requirement during the feed deprivation period.

Expression analysis using qPCR in visceral fat of the broiler and layer hens (Fig. 5c) showed that feed deprivation down-regulated expression of adipolin and *ADIPOQ* mRNAs in both broilers and layers *(P* ≤ 0.05). In broilers feed deprivation up-regulated *SFTPA1* mRNA and in layers feed deprivation down-regulated the expression of *ANGPT1, CFD and ZP3* mRNAs and upregulated *NAMPT* mRNA (*P* ≤ 0.05). Down-regulation of *ADIPOQ* mRNA by feed deprivation in chickens was shown also by Maddineni, et al., [46], but this response, as well as that of adipolin and *NAMPT*, are at an opposite direction in mammals [11, 47, 48]. It will be of high interest to understand the physiological meaning of adipolin*, ADIPOQ, ANGPT1* and *CFD* downregulation by feed deprivation while they are expressed at a higher level in LL-Br compared to FL-Br (adipolin, *ADIPOQ* and *ANGPT1*) or in LG-Br compared to HG-Br (*CFD*). In contrast, the elevation of *NAMPT* mRNA by feed deprivation is compatible with its higher mRNA expression level in LG-Br compared to HG-Br and suggests a role in restricting body growth.

The expression analysis by qPCR of the selected fat secreted proteins in broilers and layers (Fig. 5c) confirmed the differential expression between broilers and layers of adipolin, *CFD*, *LCAT*, *LECT2*, *RBP4*, *SFTPA1* and *ZP3* observed in the RNA-seq experiment (Fig. 3a). Furthermore, the qPCR experiment that was conducted on a larger number of birds (*n* = 7 per treatment), revealed an additional differential gene product, *APLN*, which were higher in layers (Fig. 5c; *P* ≤ 0.05). Interestingly, *APLN* is a known adipokine implicated in coagulation, insulin resistance and diabetes [49] and its higher expression in layers is consistent with the pattern of another pro-inflammatory adipokine in mammal, *RBP4* [50]*.*

In summary, expression of adipolin*, ADIPOQ, ANGPT1, CFD, NAMPT SFTPA1 and ZP3* mRNAs responded to feed deprivation. *NAMPT, SFTPA1 and ZP3* mRNAs were reduced by feed deprivation in either broilers (*SFTPA1*) or layers (*NAMPT and ZP3*), compatible with *NAMPT* and *SFTPA1* higher expression level in LG-Br compared to HG-Br, and with *SFTPA1* higher expression in LL-Br compared to FL-Br (Fig. 3). Thus suggesting a role of these gene products in low growth and lean phenotype. It is of high interest that feed deprivation down-regulated the mRNA expression levels of adipolin, *ADIPOQ*, *ANGPT1*, which were expressed at higher levels in LL-Br compared to the FL Br, and *CFD*, which had higher mRNA level in LG-Br compared to HG-Br. This result suggests that adipolin, *ADIPOQ*, *ANGT1*, and *CFD* are not implicated in signaling the amount of energy stores.

### Mass spectrometry (MS) analysis

MS analysis is quantitatively limited compared to RNA-seq, but provides a direct evidence of protein expression. Therefore, we investigated whether or not MS analysis of visceral fat would provide an additional perspective on the activity of the adipose tissue. Three individual broilers and three layers, grown as described in Fig. 1, were sampled at the first week of egg-lay. Using MS analysis on extracts of visceral fat, we identified 422 proteins, of which 203 showed differential expression between broilers and layers (*P* ≤ 0.05; Additional file 3: Table S5). Among the differential proteins, 27 were higher in broilers and 176 were higher in layers (Additional file 4: Fig. S1). Pathway analysis using Ingenuity software based on the significantly differential proteins (*P* ≤ 0.05) showed enrichment in broilers of the intrinsic prothrombin activation pathway (Z-score = 1, *P* < 0.0001; Additional file 3 Table S6; Additional file 4; Fig. S1). The enrichment stemmed from the higher abundance of FGA, FGB, and COL18A1 in broilers and higher COL3A1 in layers, representing only 10% of the proteins in this pathway. This result pointed to a higher coagulation activity in broilers, which is associated in mammals with atherosclerosis, inflammation and insulin resistance [51, 52], and in broiler chickens with “wooden breast disease” [53]. Enrichment of the coagulation pathway in visceral fat of chickens has been reported by Resnyk et al., based on RNA-seq analysis in LG-Br compared to HG-Br [30] and in LL-Br compared to HF-Br [29]. However, the lack of agreement between the MS and RNA-seq expression patterns for the relevant genes in this pathway (Additional file 3: Table S6B) indicated that for the expression of these genes, processes affecting translational and/or post-translational modifications have a critical impact.

To estimate the correlation between the differential patterns observed by MS and RNA-seq, we compared the respective values of log2 fold-change and found no correlation: *r*^*2*^ = 0.03, for all the gene products identified by MS and *r*^*2*^ = 0.06, for only the differential MS proteins (*P* ≤ 0.05). It is likely that in addition to variation between the two types of analysis originated from post-translational modifications, the lower depth of the MS analysis also contributed to the low correlation. Complete contradictions between the MS and RNA-seq analyses was observed only for two gene products, apolipoprotein B-100 (*APOB*), which mediates internalization of LDL particles into cells [54] and fibrinogen beta chain (*FGB*), which is an integral part fibrin matrix formation, tissue regeneration and coagulation [55]. Both these genes were expressed at a lower mRNA level in broilers compared to layers (FDR ≤ 0.05), but found at a higher level in broilers by MS (*P* ≤ 0.05). Higher plasma APOB is implicated in obesity, insulin resistance, inflammation and polycystic ovary [51, 54] and may relate to the excess of ovarian follicles in broiler hens under free feeding. Therefore, the observed higher APOB and FGB abundance in broiler compared to layers may help to explain the metabolic challenges in broiler breeders [4], and may suggest new strategies for their improvement.

**Fig. 6:**
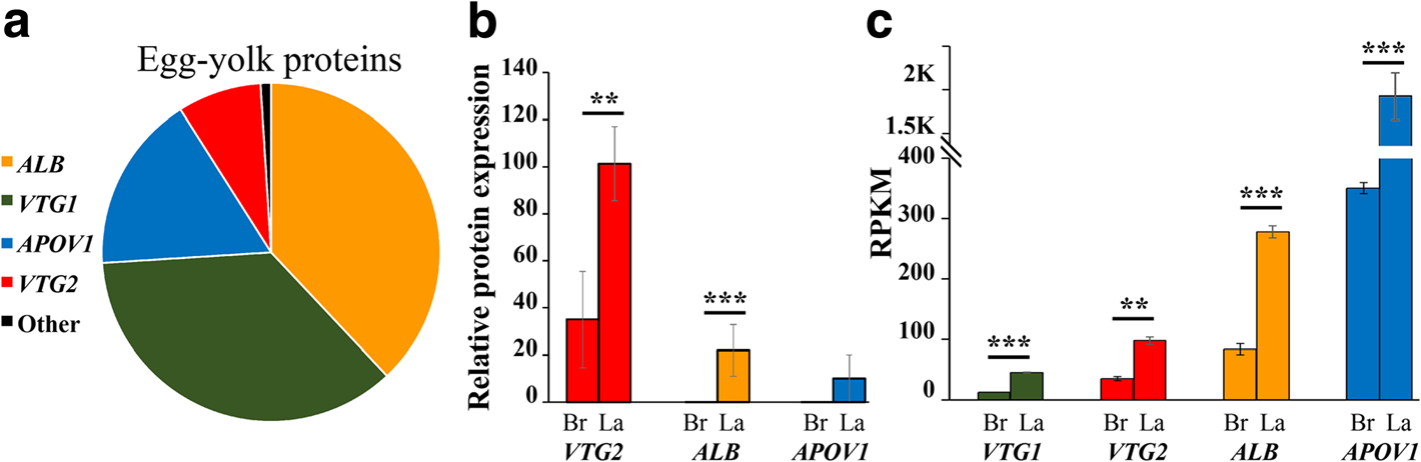
Expression of genes coding for egg-yolk proteins in visceral fat of chicken. **a** Diagram showing relative abundance of yolk proteins in chicken egg [61]. **b** Expression profile of yolk proteins identified by MS in visceral fat of broilers (Br) and layers (La) chickens. **c** Expression profile of yolk proteins’ mRNAs, detected in our RNA-seq experiment (Additional file 1: Table S2)

Among the adipokines described in Figs. 3 and 6, ADIPOQ, CFD and SERPINE2 were identified by the MS analysis, indicating their relatively high protein level. While ADIPOQ was expressed at a higher protein level in broilers, CFD and SERPINE2 were higher in layers (*P* ≤ 0.05). Among these adipokines only CFD showed a similar differential pattern also at the mRNA level (FDR = 0.05), indicating that gene expression of *ADIPOQ* and *SERPINE2* is regulated also at the translational/post translational levels. The differential pattern of ADIPOQ at the protein level is consistent with the higher adipolin mRNA expression in broiler, since both these gene products are prominent anti-inflammatory adipokines in mammals. Interestingly, both of these gene products responded similarly to feed deprivation and were enriched in LL-Br compared to FL-Br, at the mRNA level.

Another observation by MS, which was not observed at the mRNA level, was the higher-level of perilipin 1 (PLIN1) in layers and PLIN-3 in broilers (*P* < 0.005). These two lipid-droplet proteins replace one another in coating the lipid droplet, but only PLIN1 has the capacity of protecting the droplet lipids from lipolysis [56]. Therefore, this finding suggests a lower lipolysis activity in layers than in broilers under free feeding regiment. Elevated mRNA expression of *PLIN1* is chickens has been reported by Resnyk et al., in FL-Br compared to LL-Br [29], suggesting that selection for a higher *LPIN1* expression promoted the higher fat accumulation in the FL-Br. It is likely that for the commercial broiler line, the higher feed efficiency compared to layers relays on improved feed digestibility [57] rather than protection against lipolysis, suggesting a mean of further improvement of feed efficiency in broilers.

The lower sensitivity of the analysis by MS compared with mRNA data directed the focus on high abundant proteins in the visceral fat of broilers and layers. Among these proteins, we found egg-yolk proteins, which are expressed predominantly in the liver of oviparous species such as chickens (e.g. [58, 59]), but in non-avian oviparous species they were reported also in the adipose tissue [60]. Out of the four major egg yolk proteins, vitellogenin-1 (VTG1), vitellogenin-2 (VTG2), albumin (ALB), and apovitellenin-1 (APOV1) (Fig. 6a; [61]), two were detected by MS at higher expression in layers (VTG2 and ALB, *P* ≤ 0.01; Fig. 6b). Our data from the RNA-seq analysis showed higher expression (FDR ≤ 0.05) in layers for *VTG1, VTG*2, *ALB* and *APOV1* (Fig. 6c). Despite the higher number of ovarian follicles in broilers (Fig. 1), a lower production of the egg-yolk proteins was observed in their adipose tissue. These new finding of substantial expression of egg yolk proteins and mRNAs in visceral fat of chickens, together with additional mRNAs coding for the reproduction related proteins (*LCAT*, *ZP1* and *ZP3),* suggest a stronger direct cross talk between the adipose tissue and the reproduction system in chickens than previously thought.

## Discussion

The comparison of gene expression in visceral fat of commercial layer and broiler chickens under normal conditions, as well as when exposed to feed withdrawal, shed new light on the function of adipose tissue and its evolution in chickens. Pathway analysis based on RNA-seq data showed higher response to signaling in the adipose tissue of layers and elevated expression of genes in the PTEN pathway in broilers. Similar analysis on the MS results indicated enrichment in broilers of intrinsic prothrombin activation pathway implicated in coagulation and inflammation. These characterizations at the mRNA and protein levels may explain the metabolic challenges of broiler breeders when grown under free feeding, which enforces the need for a strong feed restriction in the broiler breeder industry.

**Fig. 7:**
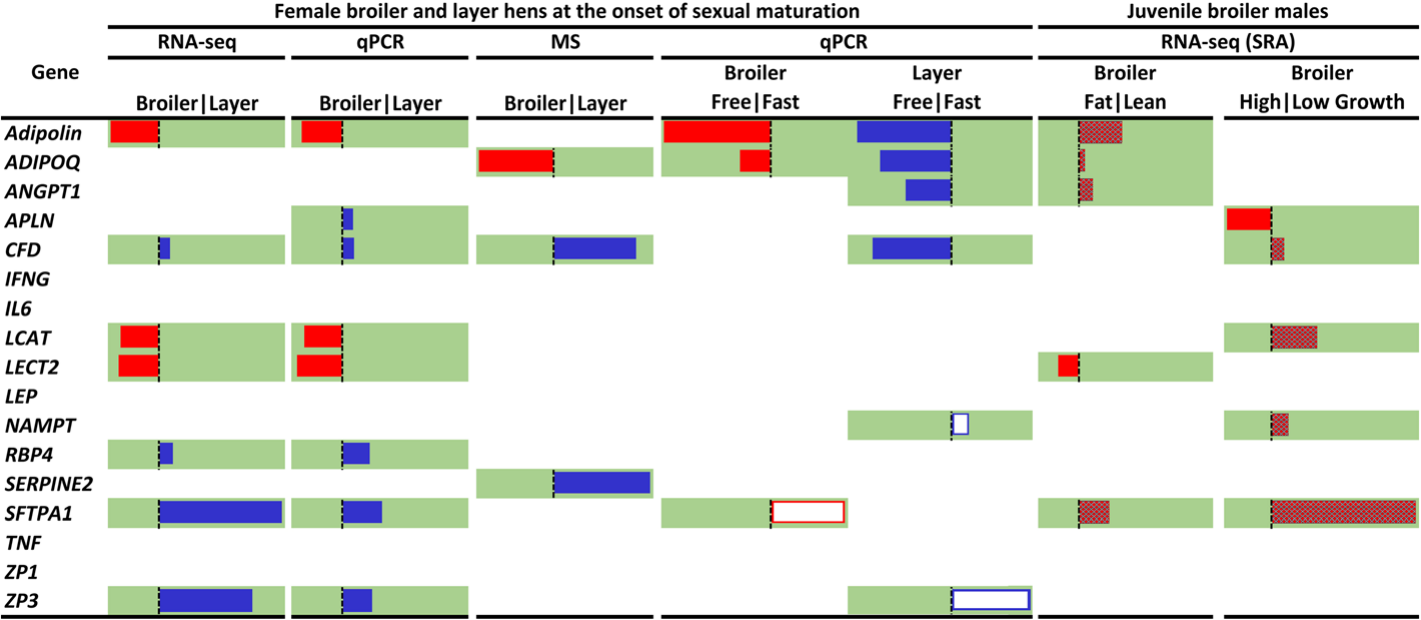
Summary of expression analyses of selected fat secreted proteins, based on values of –log[fold change] obtained in the experiments described in Figs. 3 & 5 and Additional file 3: Table S5. Green background indicates statistical significance (FDR / *P* ≤ 0.05). Red color indicates higher expression in mature broilers and juvenile fat and high-growth lines. Blue color indicates higher expression in mature layers. Empty red and blue boxes represent feed deprived mature broilers and layers, respectively. Doted red color represent juvenile lean line and low-growth line broilers. The figure was modified from Excel (Conditional Formatting)

Comparisons of our data with available RNA-seq experiments on visceral fat with broiler lines selected for high and low visceral fat accumulation or for high and low body weight provided a broader perspective for the study as summarized in Fig. 7. This summary shows additional evidence to those described before [18] confirming that the chicken leptin is not involved in signaling from visceral fat. To our surprise, three additional prominent adipokines in mammals, *TNF*, *IFNG* and *IL6* were also lowly expressed in visceral fat of the physiologically different chicken lines and following feed deprivation of broiler and layer females. Since these adipokines in mammals affect appetite, inflammation and insulin resistance, it is likely that the adipose tissue in chicken is not as critically involved in these endocrine regulatory routes as it is in mammals. Although we cannot exclude the possibility that different key genes and pathways than those operating in mammals, are effective in the endocrine regulation in chickens. In this regard, it is also important to note that three additional prominent adipokines in mammals: resistin (*RETN*), omentin (*ITLN1*) and plasminogen activator inhibitor-1 (*PAI-1*), which are implicated in the control of insulin resistance and inflammation are missing from the chicken genome assembly [14], and were not identified by our thorough search in RNA-seq data of adipose tissue [19]. Our suggestion of a limited implication of chicken visceral fat in the regulation of energy balance in chicken compared to mammals is further supported by our observation that the excess of ovarian follicles in broilers is not related to obesity. In contrast, in mammals the polycystic ovary is associated with the obesity syndrome and an excess of inflammatory adipokines [62, 63].

The expression profile of known adipokines in mammals expressed in the chicken visceral fat, including *adipolin*, *ADIPOQ*, *ANGPT1*, *APLN*, and *CFD*, did not coherently reflect the amount of fat stores. Among them, the two most prominent anti-inflammatory adipokines in mammals, adipolin and *ADIPOQ*, were expressed at a higher level in broilers than in layers (on the mRNA or protein level, respectively), and also in LL-Br compared to FL-Br. However, adipolin and *ADIPOQ* responded to feed deprivation at an opposite direction than their mammalian orthologs [11, 47, 48], as they were down- instead of up-regulated. Similarly, the proangiogenic *ANGPT1,* and the proadipogenic *CFD*, were expressed at a higher mRNA level in the LL-Br compared to FL-Br or in the LG-Br compared to HG-Br, respectively, but were down-regulated by feed deprivation. *APLN* was expressed at a higher mRNA level in layers compared to broilers and in HG-Br compared to LG-Br. This expression pattern is inconsistent with a possible implication in regulation of body growth and appetite. Similarly, CFD, which was expressed at a higher levels in layers compared to broilers (both at the mRNA and protein level) and in LG-Br compared to HG-Br (at the mRNA level), was unexpectedly downregulated by feed deprivation. *RBP4* was expressed at a higher mRNA level in layers compared to broilers, but was not dynamically expressed in the juvenile broiler lines, where it was lowly expressed (RPKM < 1; Fig. 4), suggesting its association with sexual maturation. Among the known adipokines in mammals, only expression of *NAMPT* seem constituent with energy stores as it was elevated by layer feed deprivation and in LG-Br. However, this correlation is limited since *NAMPT* was not differentially expressed in the LL vs. FL-Br, and did not respond to feed deprivation in broilers. Altogether, the expression profiles of the known adipokines in mammals that were dynamically expressed in visceral fat of chickens, seem not to report the amount of fat stores.

Some of the newly identified adipokines and fat secreted proteins (*LCAT, LECT2, SERPINE2*, *ZP1*, *APOV1, VTG1* and *VTG2*), did not respond to feed deprivation in the broiler and layer females. The expression profile of *LCAT, LECT2, SERPINE2*, *SFTPA1*, *ZP1*, *ZP3* mRNAs in red junglefowl tissues confirmed the recruitment of *LCAT*, *LECT*, and *ZP1* to visceral fat of mature female chickens and suggested that *SERPINE2*, *SFTPA1* and *ZP3* were recruited to expression in visceral fat by the selective breeding of the commercial chicken lines. For *SERPINE2* expression in visceral fat of layers was indicated also by MS and for *SFTPA1,* the high mRNA expression in layers (188 RPKM) was confirmed by its high expression also in LG-Br compared to HG-Br and in LL-Br compared to FL-Br. This dynamic expression profile of *SFTPA1* mRNA was consistent with low growth rate and low fat accumulation. The possibility of a metabolic role of SFTPA1, in addition to its known critical function in the alveoli of the lung [64], was recently suggested by Rodgaard et al. [65], showing its upregulation at the mRNA level in the liver of obese pigs. Altogether, despite the known capacity of SFTPA1 to bind phospholipids in the pulmonary alveoli [43], understanding the possible role of SFTPA1 in visceral fat of the commercial chicken lines remains an interesting challenge.

The new finding of the reproduction related proteins *ZP1*, *ZP3, APOV1, VTG1* and *VTG2,* in chicken visceral fat, suggests a more direct cross talk between the adipose tissue and the reproductive system in chickens compared to mammals. The mRNA expression pattern of adipolin, *LCAT*, *LECT2* and *RBP4* also suggest their implication in regulation of reproduction. This is based on their lower expression in the juvenile broiler lines compared to sexually mature broilers, and lower expression of *LCAT* and *LECT2* in the visceral fat of male compared to the female red junglefowl.

Recent studies have shown that the correlation, between mRNA and protein expressions can be low due to translational/post-translational modifications [60]. Nevertheless, in the current study, MS analysis indicated the recruitment of egg yolk protein expression to visceral fat of chickens and their higher expression level in layers compared to broilers, similarly to the indication found by RNA-seq. The MS analysis also indicated higher level of CFD protein in layers compared to broilers as found also at the mRNA level. An important contribution of the MS analysis was the indication of a higher ADIPOQ, SERPINE2, ABOB and FGB expression level in broilers, which were either non differential (ADIPOQ, SERPINE2) or higher in layers (ABOB and FGB) at the mRNA level. This confirmed the general conclusion that broilers express mixed markers of lean and fat phenotypes compared to layers and cannot be regarded as a model of obesity, even at ad libitum feeding. By comparing the relative accumulation of visceral fat in the two strains, we confirmed that the current broiler line does not accumulate higher relative amount of visceral fat compared to layers.

## Conclusions

Our study shows specific evolutionary adaptations of gene expression in visceral fat of chickens towards a more direct interaction with the reproductive system. The central role of the mammalian adipose tissue in the control of appetite, inflammation and insulin sensitivity seems to evolve differently in mammals and chickens. It is possible that different gene products and molecular pathways mediate this regulation in visceral fat of chickens. However, it is also possible that along the evolution of chickens/avian species the endocrine control of reproduction by the adipose tissue was more critical for survival than the control on other aspects of energy balance.

## Methods

### Animals and tissue sampling

Female broiler (Cobb) and White Leghorn (Lohman) chickens were purchased from commercial husbandries (Brown & Sons and Hasolelim, Israel, respectively) at the age of 1 day and raised in the Volcani Center in Israel, according to recommended husbandry and feeding conditions (NRC 1994) with free access to food and water (*n* = 50 for each strain). Maintenance conditions and feeding formulas for both strains were according to the Lohman guideline (http://www.hylinena.com/userdocs/products/lohmann_brown_lite_commercials_2011.pdf). This contributed to the uniformity in the experiment and is justified by the fact that the commercial formula for broiler breeders (more condensed with proteins and fat) was optimized for feed restricted regiment and may not suit free feeding. Three similar experiments were performed: Two of the experiments ended when 10 out of the 50 reached sexual maturation (indicated by egg lay, during 4 month of age), samples of abdominal (visceral) fat, hypothalamus, and pituitary were snap-frozen in liquid nitrogen after neck dislocation. Samples collected in the first experiment were used for RNA-seq analysis and samples from the second experiment were used for qPCR and MS. The third experiment ended when 10 birds from each strain reached 1 kg BW (3 weeks for broilers, 11 weeks for layers) and samples were used for measuring the relative amount of visceral fat described in Fig. 1D. At the end of the experiments, the birds were killed by neck dislocation with no use of anesthetic or euthanasia methods. All animal procedures were carried out in accordance with the National Institutes of Health Guidelines on the Care and Use of Animals and Protocol IL536/14, which was approved by the Animal Experimentation Ethics Committee of the Agricultural Research Organization, Volcani Center, Bet-Dagan, Israel.

### RNA-seq

Total RNA was prepared using a RNA isolation kit (miRNeasy, Qiagen). Libraries of cDNA were prepared by the Uppsala sequencing platform from 1 μg RNA using the TruSeq Stranded mRNA Sample Preparation Kit (Illumina Inc., #15031047 Rev. E). The libraries were uniquely tagged and sequenced with Illumina HiSeq, producing about 64 million paired-end reads (2 × 124 bp) in two technical replicates, per library (S1 Table).

### Bioinformatic analysis RNA-seq analysis

RNA-seq was performed as previously described [19]. Briefly, Trim Galore! (http://www.bioinformatics.babraham.ac.uk/projects/trim_galore) and Trimmomatic [66] were used to remove adaptor sequences and low-quality base calls from the sequencing reads. Firstly, Trim Galore! 0.3.7 was used with the parameters “-q 15 -e 0.1 -O 6 --length 36”. The second replicate was further quality-trimmed using Trimmomatic 0.32 with the parameters “SLIDINGWINDOW:10:30 LEADING:5 TRAILING:5 MINLEN:36” to remove regions of low-quality base calls. Resulting single-end reads were removed before further analysis. The quality of the resulting reads was verified using FASTQC (http://www.bioinformatics.babraham.ac.uk/projects/fastqc). Alignment was done to galGal4 using GSNAP and gene expression was quantified using featureCounts and Ensembl release 79. The two technical replicates were averaged and differential expression analysis was done using the GLM method in edgeR and an FDR threshold of 0.05.

The search for gene expression in the short read archive (SRA) database was performed using 3 RNA-seq experiments: (*i*) The ChicksPress project of red junglefowl (http://geneatlas.arl.arizona.edu/sra_data.php; [accession number ERA252218]), which includes 137 RNA-seq experiments from a male and a female red junglefowl at the age of 2 years from variety of tissues. (*ii*) Data of visceral fat from 7 weeks old broiler chickens divergently selected for fatness or leanness at the same BW (https://www.ncbi.nlm.nih.gov/geo/query/acc.cgi?acc=GSE42980; [Accession no. GSE42980] [29], obtained after 30 generation of selection with 3 biological and 2 technical replicates. (*iii*) Data of visceral fat from 7 weeks old broiler chickens divergently selected for high and low growth rate (https://www.ncbi.nlm.nih.gov/geo/query/acc.cgi?acc=GSE49121; [accession no. GSE49121]; [30]). Accession numbers of sequences used as bait for blast search in RNA-seq experiments were: GenBank: *APLN*, XM_015278386; *IFNG*, GQ421600; leptin (*LEP)*, LN794246 [18]; *TNF*, PRJEB13623 [19], *SFTPA1*, NM_001039166; *ZP3*, AB031033 *ZP1*, NM 204683; *LCAT*, NM_001293094; *LECT2*, NM_205478; *SERPINE2*, XM_015276900 and *RBM28* [19].

### Pathway analysis

Ingenuity Pathway Analysis (IPA; Qiagen, Valencia, CA; https://www.qiagenbioinformatics.com/products/ingenuity-pathway-analysis) software was used for canonical pathway analysis. Only genes showing differential expression with *q* < 0.05 (FDR corrected *P* values), total FPKM > 2, and absolute fold change > 1.5 were included.

### MS analysis

Proteomics analysis was performed based on visceral fat from each of three individuals of each chicken strain. The sample preparation and mass spectrometry has been described previously [19] and the subsequent analysis was done similarly. In short, a protein fasta database was constructed using all annotated protein-coding genes and a spectrum matching method was employed to identify significant protein hits as described previously using a randomized version of the fasta as background [19].

### QPCR

Total RNA was prepared using a RNA isolation kit (miRNeasy, Qiagen) and cDNA was synthesized from 1 μg of total RNA using the Roche First Strand Synthesis Kit. QRT-PCR was performed using StepOnePlus Real-Time PCR System (Life Technologies and the Fast SYBR Green Master Mix (Life Technologies). All samples were analyzed in biological triplicates and technical duplicates. For several genes two different sets of primers were used (Additional file 2: Table S4), and gave similar results. Transcript levels were normalized to hydroxymethylbilane synthase (*HMBS*) and for ribosomal protein 17 (not shown), which gave similar results for all the tested mRNAs.

### Statistical analysis

Statistical analyses of the RNA-seq was done using EdgeR and the false discovery rate (FDR) adjusted *P*-value using the Benjamini & Hochberg procedure, which is implemented in multtest package used for the analysis. qRT-PCR analyses were performed by one-way ANOVA and student’s t-test significant difference test (*P* ≤ 0.05). Induction of ZP1 mRNA expression upon sexual maturation (15.1 folds) was reported also by Bourin et al in the liver of layer type chickens (15.1 folds; [67]).

## Additional files

Additional file 1: RNA-seq. Data. **Table S1.** Information about the RNA sequencing. **Table S2:** Transcripts identified by RNA-seq in visceral fat of broiler and layer females at the onset of sexual maturation. **Table S3** Enriched pathways obtained using Ingenuity software and the RNA-seq differential transcripts (FDR ≤ 0.05; absolute fold change ≥1.5). **A**. List of enriched pathways. **B**. Schematic presentation of the enriched pathways. **C**. Expression pattern of the differentially expressed transcripts (FDR ≤ 0.05; absolute fold change ≥1.5) implicated in the in the *PTEN* pathway. Excel Worksheet xlsm 3.1 MB. (XLSX 3193 kb)

Additional file 2: **Table S4.** The primers used for the qPCR analyses. Excel Worksheet xlsm 12 KB. (XLSX 11 kb)

Additional file 3: MS data. **Table S5.** Proteins identified by MS in visceral fat of broiler and layer females at the onset of sexual maturation (422 proteins). **Table S6.** **A**. List of enriched pathways obtained using Ingenuity software and the MS differential proteins (*P* ≤ 0.05). **B**. List of proteins involved in the Intrinsic Prothrombin Activation Pathway: details on their expression at the protein and mRNA levels. Excel Worksheet xlsm 216 KB. (XLSX 142 kb)

Additional file 4: Ingenuity pathway analysis of the MS data. **Fig. S1A.** Venn Diagrams showing the number of proteins with either no differential (ND) or differential expression between broilers (Br) and layers (La). *n* = 3 birds per strain, *P* ≤ 0.05, absolute fold change ≥1.5. **B.** Schematic presentation of the pathways highlighted by Ingenuity software (−log *P* value > 1.3). **C.** Schematic drawing of the intrinsic prothrombin activation pathway adapted from Ingenuity software. (DOCX 654 kb)

## Abbreviations

FDR: false discovery rate
FPKM: fragments per kilobase of transcript per million mapped fragments
Galgal: *Gallus gallus* reference genome
MS: mass spectrometry
RPKM: read per kilobase of transcript per million mapped reads
SRA: short read archive
ZP: zona pellucida
