# Supplementary material for: Comparative omics and feeding manipulations in chicken indicate a shift of the endocrine role of visceral fat towards reproduction
Source: BMC Genomics. 2018 Apr 26;19:295. doi: 10.1186/s12864-018-4675-0 (PMC5922311; doi:10.1186/s12864-018-4675-0)
Supplement: Supplementary file 4 — Ingenuity pathway analysis of the MS data. Fig. S1A. Venn Diagrams showing the number of proteins with either no differential (ND) or differential expression between broilers (Br) and layers (La). n = 3 birds per strain, P ≤ 0.05, absolute fold change ≥1.5. B. Schematic presentation of the pathways highlighted by Ingenuity software (−log P value > 1.3). C. Schematic drawing of the intrinsic prothrombin activation pathway adapted from Ingenuity software. (DOCX 654 kb) [file 12864_2018_4675_MOESM4_ESM.docx]

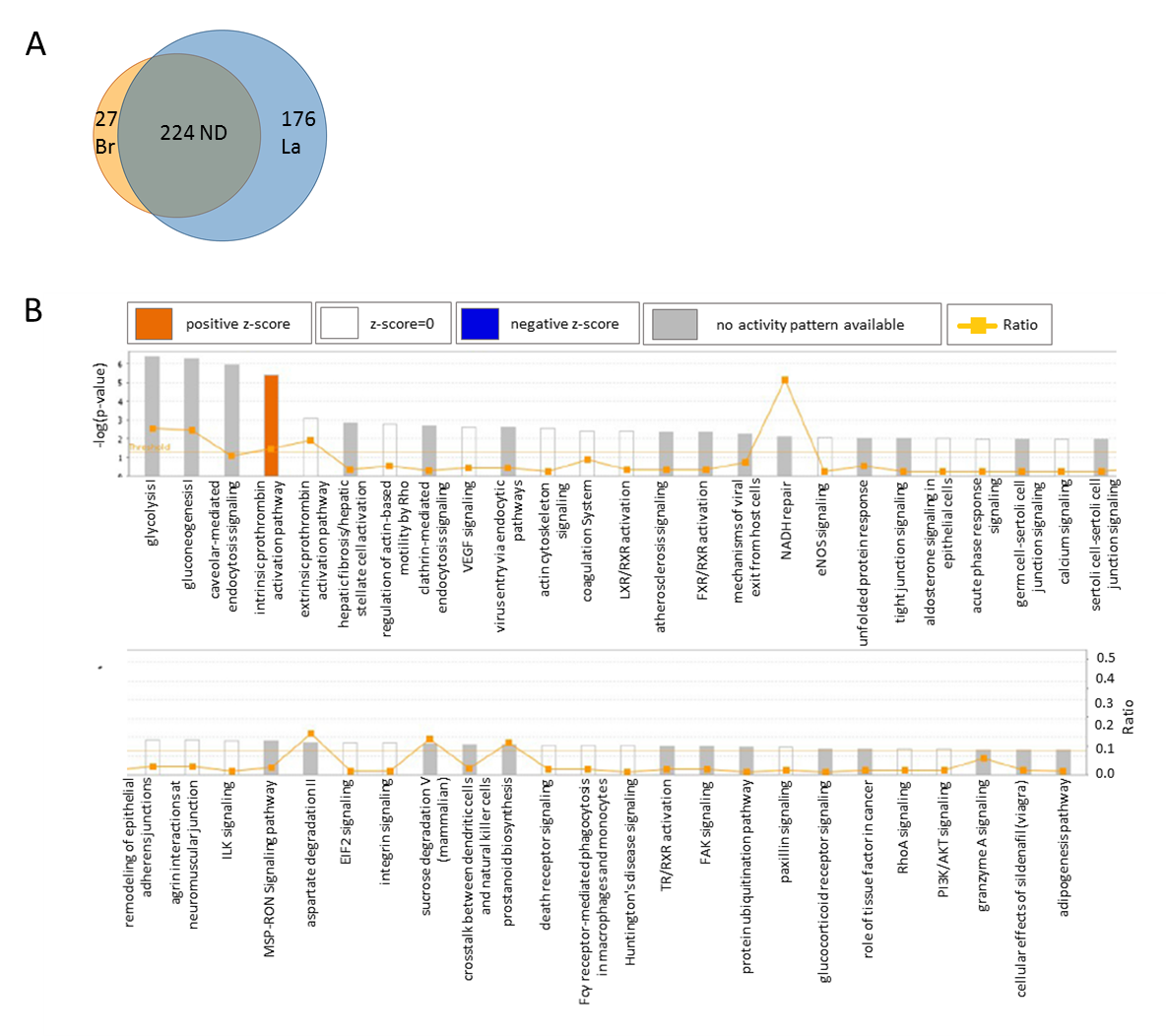
Additional File 4


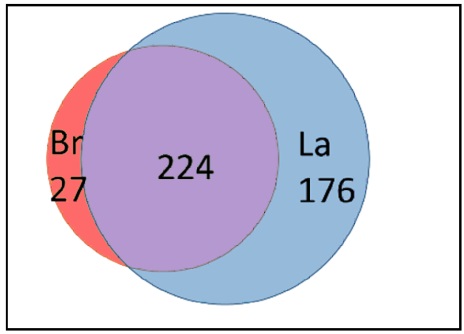


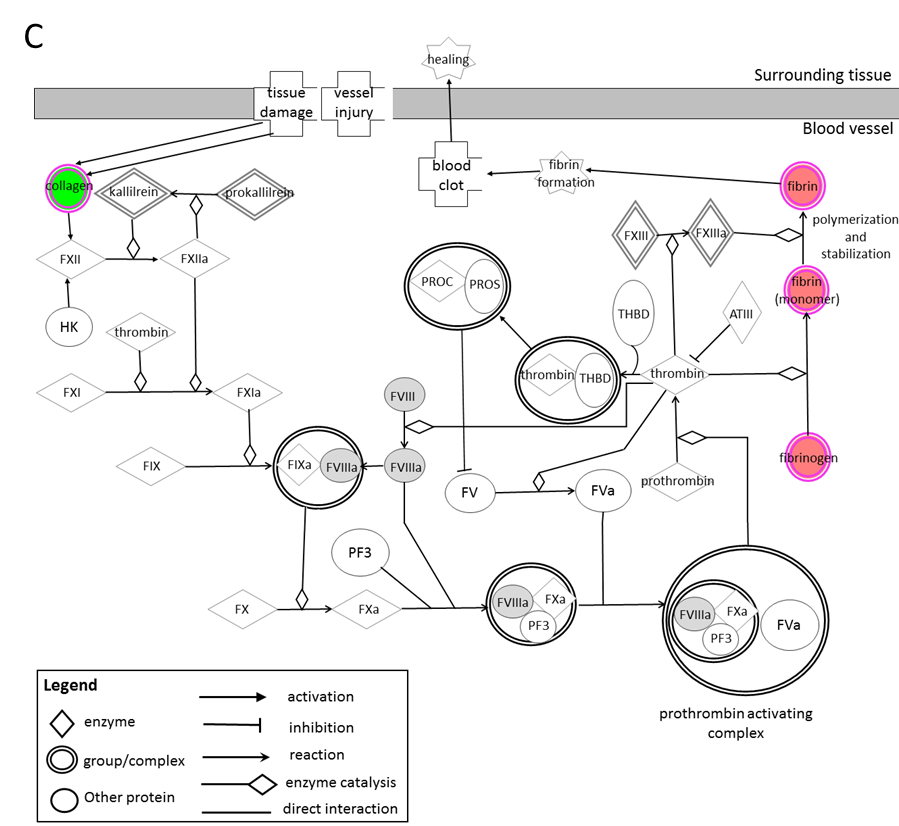


**Figure S1:** Analysis of the differential gene expression detected by MS. **a**. Venn Diagram showing the number of proteins with differential expression between broilers (Br) and layers (La), n = 3 birds per strain, *P* ≤ 0.05. **b.** Schematic presentation of the pathways highlighted by Ingenuity software (-log *P* value > 1.3). Ratio measures the overlap, meaning the ratio between the differential transcripts and the total number of transcripts implicated in the pathway (*e.g.*: 0.1 means that 10% of the genes in the pathway were differential). The overlap *P* value indicates whether there was a statistically significant overlap between the dataset's transcripts and the transcripts that were differentially expressed (calculated using Fisher’s Exact Test; significance is attributed to *P* values < 0.01). Z‐score indicates the activation states of the pathway, determined taking into account both the up or down regulation of the differential transcripts and information from the literature about the role of these gene products in the pathway (inhibition or activation). **c**. Schematic drawing of the intrinsic prothrombin activation pathway adapted from the Ingenuity software. Shapes filled in grey, green and red represent: not differential, significantly higher in layers and significantly higher in broilers, respectively.
